# Association between video consultation use and general practitioner and practice characteristics: a cross-sectional survey in Denmark

**DOI:** 10.3399/BJGPO.2024.0228

**Published:** 2025-07-02

**Authors:** Thim Prætorius, Eskild Klausen Fredslund, Daniel Cæsar Torp, Annelli Sandbæk

**Affiliations:** 1 Steno Diabetes Center Aarhus, Aarhus University Hospital, Aarhus, Denmark; 2 Department of Public Health, Aarhus University, Aarhus, Denmark

**Keywords:** general practitioners, video consultations, surveys and questionnaires, general practice, telehealth

## Abstract

**Background:**

Video consultations disrupt how general practice provides care and how patients receive it. A step towards understanding the use of video consultation is to study the association between user status and GP and practice characteristics.

**Aim:**

To study the association between GP and general practice characteristics and video consultations user status (user, never user, and former user).

**Design & setting:**

An anonymous, web-based, cross-sectional survey was distributed to all 1674 Danish general practices (single-handed, collaborative, and partnership forms) contracting with and working on a collective agreement with the public funder.

**Method:**

Multinomial logistic regression was used to correlate video consultation user status and (1) general practice characteristics, and GPs’ (2a) objective characteristics and (2b) subjective attitudes towards video consultations and organisational change.

**Results:**

The study sample included 416 GPs. Comparing users of video consultations with never users, operating as a partnership practice (relative risk ratio [RRR] 0.22; 95% confidence interval [CI] = 0.06 to 0.85; P<0.05) and practices with six or more practice staff (RRR 0.05; 95% CI = 0.01 to 0.28; P<0.001) were significantly more likely to be users. GPs with a high degree of tech savviness (RRR 0.02; 95% CI = 0.001 to 0.17; P<0.001) and openness to organisational change (RRR 0.26; 95% CI = 0.08 to 0.85; P<0.05) were significantly more likely to use video consultations.

**Conclusion:**

Characteristics of general practice and GPs are associated with video consultation user status (being a user, never user, or former user). Future research should use a difference-in-difference study design and register data to make causality claims.

## How this fits in

Video consultations in general practice represent a disruptive technology that calls for exploring who do (not) use the technology, but there are a lack of quantitative studies in this area. The present study adds to the knowledge base. It found that partnership practices and practices with more practice staff had the highest proportion of video consultation users. There was a higher likelihood of being a video consultation user with higher levels of tech savviness and openness to organisational change. If decision makers want to support use, the analysis suggests focusing on single-handed general practices, those with relatively few practice staff, and GPs with low tech savviness and organisational change openness.

## Introduction

To manage demographic changes and the growing number of people living with chronic conditions,^1^ healthcare systems encourage using healthcare technologies because they can improve clinical quality and productivity, reduce care costs, and strengthen care access and coordination.^2–5^ Internationally, the COVID-19 pandemic accelerated experimentation with models of virtual care^6^ and made GPs try out video consultations as a new way to deliver patient care.^7–12^ The push for using video consultations remains high,^13,14^ but widespread use has not yet been realised.^15^ Being a disruptive technology, video consultations challenge routine use because it changes how GPs provide patient care.^16–20^ An important step towards understanding video consultation use is to explore the characteristics of GPs and general practices who (do not) use the technology,^21,22^ but this knowledge base remains limited on such quantitative studies. The main focus has hitherto been on GPs’ user experiences,^23,24^ thereby overlooking the large body of IT research that stresses the importance of zooming in on and measuring the association between individual and organisational characteristics and technology use.^25–27^

Most insights about video consultations used in general practice have been generated by qualitative studies finding that the degree of use is related to how GPs interpret video consultations, with higher use among those who see it as a tool to improve work conditions.^28–30^ Relatedly, a strong clinician or patient preference for using the technology,^31,32^ user training,^33^ and persistence are important drivers.^34^ Even when GPs consider video consultations useful and easy to use, it does not automatically offset the effort needed to integrate the technology into existing work routines.^35,36^ GPs report in interview studies that barriers for the use of video consultation include a concern for increases in workload^37^ or a deterioration in the doctor–patient relationship.^38–40^

Studies on the association between telehealth use and organisational variables (for example, list size and number of clinicians) report mixed findings about which GPs are more likely to use healthcare technologies.^41,42^ Surveys conducted using the influential Technology Acceptance Model have found a positive association between GPs’ perceived usefulness of a healthcare technology and their attitude towards it,^43,44^ and their intention to use video consultations.^45^ The aim of this study is to explore the association between video consultation user status and GP and general practice characteristics in the context of general practices in Denmark.

## Method

### Study setting

Denmark has a decentralised and tax-financed health system. Residents are entitled to largely free care services and are assigned a general practice of their own choice through a list system. General practice owners in Denmark are self-employed but work on contracts for the public funder, and their income is generated as a combination of fee-for-service and capitation. Three forms of practice exist: single-handed, collaborative, and partnership. In a collaborative practice, two or more providers have separate patient lists and finances, but they jointly pay for costs such as offices and practice staff. A partnership practice shares patient lists and finances. At the time of data collection, the incentive for Danish GPs to use video consultations increased during the COVID-19 pandemic because the Organisation of General Practitioners (negotiates on behalf of Danish GPs) and the Danish Regions (public procurer of healthcare services) agreed on a fee-for-service for providing video consultations to patients. Video consultations are not integrated in electronic patient record systems in general practice and must be arranged through a website and carried out using an official, free-to-use app.^15^

### Research design and data collection

Data were collected through a cross-sectional, web-based survey. The survey was administered using SurveyXact and distributed to 1674 general practices representing 3326 GPs in Denmark. All trained GPs in each general practice were encouraged to participate. To identify general practices, a list of all 1718 general practices was obtained from MedCom (a provider of Danish public healthcare IT systems) in January 2021. The survey was distributed using an electronic letter on 7 January 2021, via the Danish public electronic mailbox system (e-Boks business). The letter contained information about the study and a survey link. Participants were informed about data protection measures, anonymity of participation, and the option to be paid (276.72 DKK approximately 37 EUR or 31 GPB at the time of publication) for a maximum of 20 minutes to complete the survey. Data entry for payments was conducted in a separate survey to preserve anonymity. Two reminders were sent on 21 January 2021 and 2 February 2021. The data collection ended on 7 February 2021.

### Survey measures

The dependent variable in our analysis was the video consultation user status of GPs and general practices, categorized into three user types: users, former users, or never users. Three explanatory groups of variables were used.

Five variables on the characteristics of general practice: practice type (single-handed, collaborative, or partnership), list size (divided into quintiles), municipality urbanisation index (capital area, large city, province city, suburban, and countryside), health management region (North, Central, South, Zealand or Capital), and practice staff excluding GPs (0–1, 2–3, 4–5, ≥6).

Three variables on GPs’ individual characteristics: age (divided into 5-year intervals), gender (woman, man, other, do not want to disclose), and years of experience as GP (divided into 10-year intervals).

Two variables on GPs’ individual ratings of their tech savviness (single-item measured on 1–5 Likert scale ranging from not at all to a very high degree) and organisational change readiness in general practice^46^ (consisting of six items, for example, ’there is nothing that I really need to change about the way I do my job to be more efficient’ measured on 1–5 Likert scale). The score of readiness for organisational change was divided into quintiles in the analysis. Before distribution and to test face validity, the survey was evaluated and revised according to inputs provided by a GP from each of the five Danish regions and representing practices with different characteristics.

### Statistical analysis

The analysis strategy was two-fold. First, descriptive statistics were analysed for each of the explanatory variables and their relation to the three groups of user status. Statistically significant differences in the distributions were evaluated using Pearson’s χ^2^ test. Based on registry data from the Danish Health Data Authority the demographics of the study sample was tested to determine if it differed from the population of general practices and GPs. Data were analysed using Stata (version 18.0).

Second, a multinomial logistic regression analysis was conducted to analyse the correlation between user status and all explanatory variables. The reference group was current users of video consultations compared with never and former users. The interpretation of the results from the multinomial logistic regression model was that the relative risk ratio (RRR) of never being a user increases by 20 when the RRR estimate is 1.20.

## Results

### Sample characteristics

The final sample comprised 416 responders after excluding 41 (9.0%) incomplete responses, representing 12.5% of all 3326 GPs in Denmark. The sample comprised 315 (18.8%) Danish general practices out of a total of 1674. Of the 416 responders, 262 (63.0%) were users, 80 (19.2%) were former users, and 74 (17.8%) were never users. Pearson’s χ^2^ tests showed that the GPs’ gender and age groups in the study sample were statistically identical to the population not participating. The study sample differed from the population by having higher participation among GPs from partnership practices and larger cities (Table 1). The incomplete responses were statistically similar to those of the complete responses with respect to age and gender.

**Table 1.** Responders in sample and comparison with the remaining population

Characteristics^a^	Sample (*n* = 416) *n* (%)		Population (*n* = 2901) *n* (%)		Pearson χ^2^ (*P*-value)
Gender, woman^b^	221	(53.1)	1659	(57.1)	0.166
Age groups, years^b^					
30–39	26	(6.4)	205	(7.1)	0.819
40–44	73	(18.0)	577	(20.0)	
45–49	98	(24.2)	614	(21.2)	
50–54	57	(14.1)	416	(14.4)	
55–59	63	(15.6)	433	(15.0)	
60–64	55	(13.6)	387	(13.4)	
≥65	33	(8.2)	260	(9.0)	
Municipality urbanisation index^c^					0.019
Capital area	129	(31.0)	789	(25.5)	
Large city	63	(15.1)	392	(12.7)	
Province city	85	(20.4)	754	(24.4)	
Suburban	68	(16.4)	507	(16.4)	
Countryside	71	(17.0)	654	(21.1)	
Practice type^d^					<0.001
Single-handed	102	(24.5)	447	(35.7)	
Collaborative	52	(12.5)	145	(11.6)	
Partnership	262	(63.0)	659	(52.7)	

^a^Missing data in the sample and population means that sums do not add to the total sample size and population of GPs. ^b^Population data from Organisation of General Practitioners. ^c^Municipality types based on definition by Statistics Denmark. ^d^Population calculated from data by the Danish Health Data Authority.

### Descriptive statistics: general practice and GPs’ characteristics and video consultation user status

Table 2 shows the distribution and characteristics of general practices and GPs. The partnership form of practice had the highest proportion of users of video consultations (71.4%) compared with former users (56.2%) and never users (40.5%). In comparison with never users, there was a tendency for a higher proportion of users of video consultations among general practices with more patients and a higher number of practice staff were more prevalent users of video consultations. There was no significant gender difference across the three groups of user status. Older and more experienced GPs appeared less likely to offer video consultations. The proportion of users were higher among GPs with a high and very high degree of tech savviness (in total 48.1%) compared with former and never users. A similar inclination towards being open to organisational change was found across the three user groups.

**Table 2.** Video consultation user status and general practice and GP characteristics

	User	Former user	Never user	Total	Pearson χ^2^ (*P*-value)
*n* (%)	262 (63.0)	80 (19.2)	74 (17.8)	416 (100.0)	
Practice type, *n* (%)					
Single-handed	47 (17.9)	19 (23.8)	36 (48.6)	102 (24.5)	
Partnership	187 (71.4)	45 (56.2)	30 (40.5)	262 (63.0)	
Collaborative	28 (10.7)	16 (20.0)	8 (10.8)	52 (12.5)	0.00
List size (quintile), *n* (%)					
510–1749	33 (12.8)	20 (25.0)	22 (30.1)	75 (18.3)	
1750–3199	45 (17.5)	19 (23.8)	22 (30.1)	86 (21.0)	
3200–3799	60 (23.3)	12 (15.0)	10 (13.7)	82 (20.0)	
3800–5899	55 (21.4)	16 (20.0)	12 (16.4)	83 (20.2)	
≥5900	64 (24.9)	13 (16.2)	7 (9.6)	84 (20.5)	0.00
Municipality urbanisation index, *n* (%)					
Capital area	73 (27.9)	35 (43.8)	21 (28.4)	129 (31.0)	
Large city	51 (19.5)	7 (8.8)	5 (6.8)	63 (15.1)	
Province city	59 (22.5)	11 (13.8)	15 (20.3)	85 (20.4)	
Suburban	41 (15.6)	15 (18.8)	12 (16.2)	68 (16.3)	
Countryside	38 (14.5)	12 (15.0)	21 (28.4)	71 (17.1)	0.00
Region, *n* (%)					
North	14 (5.4)	4 (5.0)	3 (4.1)	21 (5.1)	
Central	85 (32.7)	17 (21.2)	15 (20.5)	117 (28.3)	
South	51 (19.6)	8 (10.0)	16 (21.9)	75 (18.2)	
Zealand	24 (9.2)	15 (18.8)	12 (16.4)	51 (12.3)	
Capital	86 (33.1)	36 (45.0)	27 (37.0)	149 (36.1)	0.03
Practice staff, *n* (%)					
0–2	11 (4.2)	12 (15.0)	17 (23.0)	40 (9.6)	
2–3	72 (27.5)	32 (40.0)	28 (37.8)	132 (31.7)	
4–5	70 (26.7)	20 (25.0)	20 (27.0)	110 (26.4)	
≥6	109 (41.6)	16 (20.0)	9 (12.2)	134 (32.2)	0.00
Gender, *n* (%)					
Woman	145 (55.3)	40 (50.0)	36 (48.6)	221 (53.1)	
Man	117 (44.7)	40 (50.0)	38 (51.4)	195 (46.9)	0.49
Age group, *n* (%)					
30–39	16 (6.3)	7 (9.0)	3 (4.2)	26 (6.4)	
40–44	54 (21.2)	13 (16.7)	6 (8.3)	73 (18.0)	
45–49	75 (29.4)	10 (12.8)	13 (18.1)	98 (24.2)	
50–54	38 (14.9)	11 (14.1)	8 (11.1)	57 (14.1)	
55–59	32 (12.5)	18 (23.1)	13 (18.1)	63 (15.6)	
60–64	27 (10.6)	15 (19.2)	13 (18.1)	55 (13.6)	
≥65	13 (5.1)	4 (5.1)	16 (22.2)	33 (8.1)	0.00
Years of experience as doctor, *n* (%)					
<10	108 (41.2)	26 (32.9)	21 (28.8)	155 (37.4)	
10–19	89 (34.0)	26 (32.9)	16 (21.9)	131 (31.6)	
20–29	53 (20.2)	25 (31.6)	29 (39.7)	107 (25.8)	
≥30	12 (4.6)	2 (2.5)	7 (9.6)	21 (5.1)	0.00
Years of experience in general practice					
<10	136 (52.3)	33 (41.8)	31 (43.1)	200 (48.7)	
10–19	78 (30.0)	32 (40.5)	17 (23.6)	127 (30.9)	
20–29	38 (14.6)	13 (16.5)	17 (23.6)	68 (16.5)	
≥30	8 (3.1)	1 (1.3)	7 (9.7)	16 (3.9)	0.01
Tech savviness, *n* (%)					
Not at all	3 (1.1)	1 (1.2)	3 (4.1)	7 (1.7)	
To a lesser degree	27 (10.3)	14 (17.5)	22 (29.7)	63 (15.1)	
To some degree	106 (40.5)	30 (37.5)	37 (50.0)	173 (41.6)	
To a high degree	99 (37.8)	20 (25.0)	9 (12.2)	128 (30.8)	
To a very high degree	27 (10.3)	15 (18.8)	3 (4.1)	45 (10.8)	0.00
Openness to organisational change (quintile), *n* (%)					
Very low	28 (11.2)	13 (17.1)	13 (18.6)	54 (13.6)	
Low	49 (19.5)	15 (19.7)	19 (27.1)	83 (20.9)	
Mid	52 (20.7)	16 (21.1)	11 (15.7)	79 (19.9)	
High	59 (23.5)	17 (22.4)	14 (20.0)	90 (22.7)	
Very high	63 (25.1)	15 (19.7)	13 (18.6)	91 (22.9)	0.52

### Regression analysis: users of video consultation compared with never and former users

Based on the full multinomial logistic regression model, Table 3 presents the correlation between general practice and GP characteristics and the three groups of user status.

**Table 3.** Multinomial logistic regression of video consultation user status

Reference group: Users of video consultation	Never user (RRR; CI)		Former user (RRR; CI)	
Practice type (ref: single-handed)	-		-	
Partnership	0.22^a^	(0.06 to 0.85)	3.29	(0.95 to 11.39)
Collaborative	0.48	(0.14 to 1.68)	2.61	(0.90 to 7.61)
List size (ref: 510–1749)	-		-	
1750–3199	0.70	(0.24 to 2.07)	0.42	(0.15 to 1.19)
3200–3799	1.08	(0.22 to 5.32)	0.11^b^	(0.02 to 0.50)
3800–5899	2.29	(0.46 to 11.37)	0.18^a^	(0.04 to 0.81)
≥5900	4.09	(0.60 to 27.70)	0.22	(0.04 to 1.09)
Municipality urbanisation index (ref: Capital area)	-		-	
Large city	0.37	(0.06 to 2.41)	0.48	(0.10 to 2.27)
Province city	1.05	(0.27 to 4.08)	0.54	(0.16 to 1.85)
Suburban	0.94	(0.23 to 3.80)	1.43	(0.44 to 4.64)
Countryside	1.74	(0.40 to 7.61)	0.83	(0.21 to 3.24)
Region (ref: Northern Jutland)	-		-	
Central Jutland	1.39	(0.21 to 9.35)	1.58	(0.31 to 8.11)
Southern Denmark	3.01	(0.45 to 20.15)	1.08	(0.19 to 6.26)
Zealand	1.85	(0.25 to 13.68)	4.33	(0.70 to 26.61)
Capital	2.18	(0.27 to 17.80)	2.21	(0.39 to 14.83)
Practice staff (ref: 0–2)	-		-	
2–3	0.47	(0.14 to 1.60)	1.35	(0.38; 4.80)
4–5	0.36	(0.09 to 1.50)	0.76	(0.19; 3.10)
≥6	0.05^c^	(0.01 to 0.28)	0.32	(0.07; 1.47)
Gender (ref: woman)	-		-	
Man	1.57	(0.66 to 3.76)	2.15^a^	(1.08 to 4.30)
Age group (ref: 40–44)	-		-	
30–39	0.87	(0.11 to 6.98)	2.63	(0.67 to 10.38)
45–49	2.44	(0.68 to 8.69)	0.63	(0.20 to 1.95)
50–54	4.49	(0.76 to 26.57)	1.35	(0.30 to 6.07)
55–59	4.31	(0.57 to 32.57)	3.99	(0.82 to 19.51)
60–64	3.46	(0.38 to 31.80)	7.32^a^	(1.11 to 48.44)
≥65	21.71^a^	(1.92 to 245.70)	2.92	(0.28 to 30.29)
Years of experience as doctor (ref:<10 years)	-		-	
10–19	0.52	(0.13 to 2.18)	0.36	(0.09 to 1.55)
20–29	1.31	(0.18 to 9.57)	0.40	(0.06 to 2.57)
≥30	1.15	(0.04 to 29.36)	0.14	(0.01 to 2.41)
Years of experience in general practice (ref:<10 year)	-		-	
10–19	0.41	(0.13 to 1.36)	2.86	(0.86 to 9.56)
20–29	0.18^a^	(0.04 to 0.82)	0.54	(0.12 to 2.52)
≥30	0.24	(0.01 to 4.70)	0.61	(0.02 to 18.30)
Tech savviness (ref: Not at all)	-		-	
To a lesser degree	0.61	(0.07 to 5.51)	2.66	(0.16 to 45.52)
To some degree	0.16	(0.02 to 1.36)	1.15	(0.07 to 18.11)
To a high degree	0.02^c^	(0.001 to 0.17)	0.99	(0.06 to 16.33)
To a very high degree	0.08	(0.01 to 1.07)	4.10	(0.22 to 74.63)
Openness to organisational change (ref: very low)	-		-	
Low	0.95	(0.31 to 2.93)	0.57	(0.20 to 1.68)
Mid	0.44	(0.12 to 1.56)	0.55	(0.19 to 1.62)
High	0.26^a^	(0.08 to 0.85)	0.34^a^	(0.12 to 0.99)
Very high	0.67	(0.20 to 2.27)	0.37	(0.12 to 1.13)
Observations	379		379	

*P*-values in parentheses: ^a^ *P*<0.05. ^b^ *P*<0.01. *^c^P*<0.001.

Comparing never users with users showed that having a higher number of practice staff (≥6 staff; RRR 0.05; 95% CI = 0.01 to 0.28) and operating as a partnership practice (RRR 0.22; 95% CI = 0.06 to 0.85) were associated with a lower likelihood of never using video consultations. There was a gradient towards a higher likelihood of not using video consultations with higher age, and with a significant estimate for the ≥65 years olds (RRR 21.71; 95% CI = 1.92 to 245.70; P<0.05). The likelihood of a GP being a never user declined when the level of tech savviness increased. A significant correlation was found for tech savviness to a high degree (RRR 0.02; 95% CI = 0.001 to 0.17; P<0.001). GPs with a high degree of openness to organisational change had a significantly lower likelihood of being a never user (RRR 0.26; 95% CI = 0.08 to 0.85; P<0.05).

When comparing users with former users, a larger list size (for example, for 3200–3799 patients: RRR 0.11; 95% CI = 0.02 to 0.50; P<0.01) was significantly associated with a lower likelihood of being a former user. There was a gradient towards higher probabilities of being a former user when age increased (for 60–65 year olds: RRR 7.32; 95% CI = 1.11 to 48.44). GPs with a high openness to organisational change had a lower likelihood of being a former user (RRR 0.34; 95% CI = 0.12 to 0.99) Male GPs ware significantly associated with being a former user (RRR 2.15; 95% CI = 1.08 to 4.30; ; P<0.05).

## Discussion

### Summary

Based on a web-based survey of Danish GPs, our analysis shows that users of video consultations were more likely to work in larger, partnership practices, and practices with more practice staff (≥6). The likelihood also increased when GPs had higher levels of tech savviness and openness for organisational change. Smaller general practices and practices located in more rural areas were less likely to provide video consultations. Our survey of individual and organisational characteristics contributes knowledge about explanatory variables that only have been used in a few quantitative studies surveying GPs and video consultation user status.

### Strengths and limitations

The sample was representative with respect to age and gender, but not with respect to practice size or type. The relatively small study sample of 12.5% of all 3326 Danish GPs introduces the risk of selection bias. Our results contribute insights to the scarce knowledge of to what degree individual and organisational characteristics are associated with the use of video consultations in general practice. It is possible that the study sample was more tech savvy or had stronger opinions about video consultations than the total population, which might bias the results. The composition of patients in each general practice could not be controlled for. We also note that data collection took place during the COVID-19 pandemic, where the volume of video consultations increased. This unusual backdrop of circumstances could have influenced how GPs responded to the call for providing video consultations and their willingness to participate in the survey.

### Comparison with existing literature

Prior surveys have studied user experiences of GPs^23,24^ and the association between GPs’ perceived usefulness, attitude towards and intention to use video consultations.^45^ Similar to our findings, a 2021 survey of Australian GPs on telehealth use finds an association with individual and organisational characteristics, including a higher proportion of use in larger practices and less use when GPs were aged >55 years.^21^ A survey of German GPs also finds that significant predictors of digital health adoption and future use included practice characteristics and GPs’ digital affinity and personality traits, thereby highlighting factors that should be considered when aiming to implement digitalisation in general practice.^22^

The finding that GPs with lower levels of tech savviness and organisational change readiness are less likely to be users points to two additional explanatory variables relevant for understanding the limited international use of video consultations after COVID-19.^14,15^ In Denmark, the volume increased to 2.9% compared with the pre-pandemic volume of 0.1%, but decreased to 1.2% in 2023 even with a higher fee for providing video consultations.^15^ Our study findings suggest that the increase during the pandemic and the continued use after is driven by those with a higher implementation capacity, something that our results find to be higher in larger practices with more staff and among GPs with higher levels of tech savviness and change openness.

### Implications for research and practice

Given that the decision to start using video consultations largely rests with the GP, although European health policymakers are incentivising mandatory use,^15^ our study suggests zooming in on individual characteristics that are receptive to educational and training interventions, something a recent study found to be important.^33^ If policymakers want to increase the uptake of video consultations, our findings point towards focusing on single-handed practices with fewer staff. If the information can be collected, it is relevant to reach out to general practices and practitioners with low tech savviness and lower openness to change. Decision-makers and policymakers should also be mindful of how GPs interpret video consultations^28^ and how the technology can be integrated into existing work routines.^34–36^ To develop knowledge on the use of video consultations, future research could benefit from examining the underlying mechanisms driving the study findings. One promising approach is combining the detailed data available in Danish registries^47^ with a difference-in-difference study design that opens for making causality claims^48^ between the use of video consultation and healthcare quality.

## Data

The dataset relied on in this article is not publicly available, but de-identified data will be shared upon reasonable request for the purpose of replication by contacting the corresponding author.

## References

[1] Barnett K, Mercer SW, Norbury M (2012). Epidemiology of multimorbidity and implications for health care, research, and medical education: a cross-sectional study. Lancet.

[2] Bronsoler A, Doyle J, Van Reenen J (2022). The impact of health information and communication technology on clinical quality, productivity, and workers. Annu Rev Econ.

[3] Litwin AS (2020). Technological change in health care delivery: its drivers and consequences for work and workers.

[4] Greenlee MC, Bolen S, Chong W (2023). The National Clinical Care Commission Report to Congress: leveraging federal policies and programs to improve diabetes treatment and reduce complications. Diabetes Care.

[5] NHS England (2019). The NHS long term plan.

[6] Keesara S, Jonas A, Schulman K (2020). Covid-19 and health care’s digital revolution. N Engl J Med.

[7] Greenhalgh T, Wherton J, Shaw S, Morrison C (2020). Video consultations for covid-19. BMJ.

[8] Groenewegen PP, van den Muijsenbergh M, Batenburg R (2023). Quick adaptation of the organisation of general practices during the COVID-19 pandemic in the Netherlands. BMC Prim Care.

[9] Norberg BL, Getz LO, Johnsen TM (2023). General practitioners’ experiences with potentials and pitfalls of video consultations in Norway during the COVID-19 lockdown: qualitative analysis of free-text survey answers. J Med Internet Res.

[10] Murphy M, Scott LJ, Salisbury C (2021). Implementation of remote consulting in UK primary care following the COVID-19 pandemic: a mixed-methods longitudinal study. Br J Gen Pract.

[11] Stummer FO, Voggenberger L, Gomez Pellin M de la C (2023). Insights into the use of telemedicine in primary care in times of the SARS-CoV-2 pandemic — a cross-sectional analysis based on the international PRICOV-19 study in Austria. BMC Prim Care.

[12] Kirk UB, Høstrup Vestergaard C, Hammer Bech B (2024). Video consultation in general practice during COVID-19: a register-based study in Denmark. BJGP Open.

[13] Mold F, Cooke D, Ip A (2021). COVID-19 and beyond: virtual consultations in primary care-reflecting on the evidence base for implementation and ensuring reach: commentary article. BMJ Health Care Inform.

[14] Car J, Koh G-H, Foong PS, Wang CJ (2020). Video consultations in primary and specialist care during the covid-19 pandemic and beyond. BMJ.

[15] Assing Hvidt E, Atherton H, Keuper J (2023). Low adoption of video consultations in post-COVID-19 general practice in Northern Europe: barriers to use and potential action points. J Med Internet Res.

[16] Salisbury C, Quigley A, Hex N, Aznar C (2020). Private video consultation services and the future of primary care. J Med Internet Res.

[17] Nordtug M, Assing Hvidt E, Lüchau EC, Grønning A (2022). General practitioners’ experiences of professional uncertainties emerging from the introduction of video consultations in general practice: qualitative study. JMIR Form Res.

[18] Wherton J, Shaw S, Papoutsi C (2020). Guidance on the introduction and use of video consultations during COVID-19: important lessons from qualitative research. BMJ Leader.

[19] James HM, Papoutsi C, Wherton J (2021). Spread, scale-up, and sustainability of video consulting in health care: systematic review and synthesis guided by the NASSS framework. J Med Internet Res.

[20] Kofod FG, Assing Hvidt E, Arreskov AB, Guassora AD (2024). Interpersonal contact and altered sensory conditions in video consultation — a qualitative interview study in Danish general practice. Scand J Prim Health Care.

[21] Scott A, Bai T, Zhang Y (2021). Association between telehealth use and general practitioner characteristics during COVID-19: findings from a nationally representative survey of Australian doctors. BMJ Open.

[22] Weik L, Fehring L, Mortsiefer A, Meister S (2024). Understanding inherent influencing factors to digital health adoption in general practices through a mixed-methods analysis. NPJ Digit Med.

[23] Wanderås MR, Abildsnes E, Thygesen E, Martinez SG (2023). Video consultation in general practice: a scoping review on use, experiences, and clinical decisions. BMC Health Serv Res.

[24] Thiyagarajan A, Grant C, Griffiths F, Atherton H (2020). Exploring patients’ and clinicians’ experiences of video consultations in primary care: a systematic scoping review. BJGP Open.

[25] Greenhalgh T, Wherton J, Papoutsi C (2017). Beyond adoption: a new framework for theorizing and evaluating nonadoption, abandonment, and challenges to the scale-up, spread, and sustainability of health and care technologies. J Med Internet Res.

[26] Heinsch M, Wyllie J, Carlson J (2021). Theories informing ehealth implementation: systematic review and typology classification. J Med Internet Res.

[27] Callen JL, Braithwaite J, Westbrook JI (2008). Contextual implementation model: a framework for assisting clinical information system implementations. J Am Med Inform Assoc.

[28] Lüchau EC, Atherton H, Olesen F (2023). Interpreting technology: use and non-use of doctor–patient video consultations in Danish general practice. Soc Sci Med.

[29] Hammerton M, Benson T, Sibley A (2022). Readiness for five digital technologies in general practice: perceptions of staff in one part of southern England. BMJ Open Qual.

[30] De Guzman KR, Snoswell CL, Giles CM (2022). GP perceptions of telehealth services in Australia: a qualitative study. BJGP Open.

[31] Greenhalgh T, Ladds E, Hughes G (2022). Why do GPs rarely do video consultations? Qualitative study in UK general practice. Br J Gen Pract.

[32] Van den Heuvel J, Assing Hvidt E, Dahl-Larsen R (2024). Patient-initiated video consultations: a qualitative study on reasons for patient-booking in Danish general practice. Digit Health.

[33] Greenhalgh T, Payne R, Hemmings N (2024). Training needs for staff providing remote services in general practice: a mixed-methods study. Br J Gen Pract.

[34] Lüchau EC, Olesen F, Atherton H (2024). The invisible work of video consultation use in Danish general practice: an ethnographic study. Digit Health.

[35] Hughes G, Moore L, Maniatopoulos G (2022). Theorising the shift to video consulting in the UK during the COVID-19 pandemic: analysis of a mixed methods study using practice theory. Soc Sci Med.

[36] Wanderås MR, Abildsnes E, Thygesen E, Martinez SG (2024). Hammering nails with a screwdriver: how GPs perceive video consultations. BJGP Open.

[37] Salisbury C, Murphy M, Duncan P (2020). The impact of digital-first consultations on workload in general practice: modeling study. J Med Internet Res.

[38] Hammersley V, Donaghy E, Parker R (2019). Comparing the content and quality of video, telephone, and face-to-face consultations: a non-randomised, quasi-experimental, exploratory study in UK primary care. Br J Gen Pract.

[39] Björndell C, Premberg Å (2021). Physicians’ experiences of video consultation with patients at a public virtual primary care clinic: a qualitative interview study. Scand J Prim Health Care.

[40] Donaghy E, Atherton H, Hammersley V (2019). Acceptability, benefits, and challenges of video consulting: a qualitative study in primary care. Br J Gen Pract.

[41] Saint-Lary O, Gautier S, Le Breton J (2020). How GPs adapted their practices and organisations at the beginning of COVID-19 outbreak: a French national observational survey. BMJ Open.

[42] Evans JMM, Guthrie B, Pagliari C (2008). Do general practice characteristics influence uptake of an information technology (IT) innovation in primary care?. Inform Prim Care.

[43] Kivekäs E, Enlund H, Borycki E, Saranto K (2016). General practitioners’ attitudes towards electronic prescribing and the use of the national prescription centre. J Eval Clin Pract.

[44] Holden RJ, Karsh BT (2010). The technology acceptance model: its past and its future in health care. J Biomed Inform.

[45] Torp DC, Sandbæk A, Prætorius T (2022). The technology acceptance of video consultations for type 2 diabetes care in general practice: cross-sectional survey of Danish general practitioners. J Med Internet Res.

[46] Christl B, Harris MF, Jayasinghe UW (2010). Readiness for organisational change among general practice staff. Qual Saf Health Care.

[47] Andersen JS, Olivarius NDF, Krasnik A (2011). The Danish National Health Service Register. Scand J Public Health.

[48] Wing C, Simon K, Bello-Gomez RA (2018). Designing difference in difference studies: best practices for public health policy research. Annu Rev Public Health.

